# Survival Rates of Amalgam and Composite Resin Restorations from Big Data Real-Life Databases in the Era of Restricted Dental Mercury Use

**DOI:** 10.3390/bioengineering11060579

**Published:** 2024-06-07

**Authors:** Guy Tobias, Tali Chackartchi, Jonathan Mann, Doron Haim, Mordechai Findler

**Affiliations:** 1 Department of Community Dentistry, Hadassah Medical Center, Faculty of Dental Medicine, Hebrew University of Jerusalem, Jerusalem 76841, Israel; dman@hadassah.org.il; 2 Department of Periodontology, Hadassah Medical Center, Faculty of Dental Medicine, Hebrew University of Jerusalem, Jerusalem 76841, Israel; 3 Dental Research Unit-Maccabi-Dent, Maccabi Healthcare Fund, Tel Aviv 6971028, Israel; haim_d@maccabi-dent.com (D.H.); findlermo@gmail.com (M.F.)

**Keywords:** tooth decay, big data, amalgam, composite resin

## Abstract

Tooth decay, also known as caries, is a significant medical problem that harms teeth. Treatment is based on the removal of the carious material and then filling the cavity left in the tooth, most commonly with amalgam or composite resin. The consequences of filling failure include repeating the filling or performing another treatment such as a root canal or extraction. Dental amalgam contains mercury, and there is a global effort to reduce its use. However, no consensus has been reached regarding whether amalgam or composite resin materials are more durable, and which is the best restorative material, when using randomized clinical trials. To determine which material is superior, we performed a retrospective cohort study using a large database where the members of 58 dental clinics with 440 dental units were treated. The number of failures of the amalgam compared to composite resin restorations between 2014 and 2021 were compared. Our data included information from over 650,000 patients. Between 2014–2021, 260,905 patients were treated. In total, 19,692 out of the first 113,281 amalgam restorations failed (17.49%), whereas significantly fewer composite restorations failed (11.98%) with 65,943 out of 555,671. This study indicates that composite is superior to amalgam and therefore it is reasonable to cease using mercury-containing amalgam.

## 1. Introduction

There are many techniques for restoring tooth structure that is lost due to caries. The most common procedure is a filling, using amalgam or a composite resin material. More complicated and costly procedures such as crowns, laminates, ceramic fillings, or gold or ceramic inlays, as well as, in more recent use, new materials like indirect resin restorations, glass ionomers, and new technologies combining digital workflow, are also performed.

The decision of what restoration type to use is usually made after the advantages and disadvantages of each procedure are considered: appearance, cost, length of the procedure, complications, and longevity. In addition, amalgam fillings can expand with age or undergo metal fatigue and break down, losing their seal and allowing decay to develop, whereas composite resin restorations tend to wear out sooner than metal fillings, and they may stain from frequent exposure to coffee, tea, red wine, and other foods with staining properties. All of these factors mentioned above are discussed with the patient. The provider often explains which procedure they have more experience with, and possibly which procedure involves a higher success rate.

Although providers emphasize primary prevention, this is often not achieved, and the consequences are dental decay or gum disease. To prevent the carious lesion worsening and progression toward the nerve of the tooth necessitating root canal therapy, providers use fillings as a secondary prevention technique. As mentioned, the most common restorative materials used for “simple” fillings are amalgam and composite resin.

In the United States, 100 million people have amalgam fillings, and 100 million amalgams are placed yearly [1]. According to a 2015 publication [2], dental amalgam remains a predictable, cost-effective, and safe means for the restoration of posterior teeth. It is important to note that amalgam has been used for the last 150 years, where only gold alloys have been used in the restoration of teeth for longer [3]. Alloys of mercury with metals such as silver, copper, tin, and zinc [4] have been used in restorative material amalgam [5]. Dental amalgam has been studied and reviewed extensively, and it has an established record of safety and effectiveness [6]. An FDA 2004 report stated that “The current data are insufficient to support an association between mercury release from dental amalgam and the various complaints that have been attributed to this restoration material” [7]. In an article in JAMA from 2006 [8], the authors concluded that “there were no statistically significant differences in adverse neuropsychological or renal effects observed over the 5-year period in children using dental amalgam or composite materials”. In 2009, the FDA literature supported the position that “amalgam is a valuable, viable, and safe choice for dental patients” [9].

On the other hand, at the 2013 Minamata Convention [10] (named after the bay in Japan where in the mid-20th century, mercury-tainted industrial wastewater poisoned thousands of people), the most recent global agreement on environment and health was reached. A decision was made to reduce all forms of mercury use. One of the outcomes of this decision adopted in the US was to phase-down dental amalgam use by increasing the use of other restorative materials [11]. Concerns have also been raised about the potential toxicity of composite resin compounds [12].

The superior longevity of amalgam has been demonstrated in several publications [13,14]. A meta-analysis from 2016 [15] concluded that composite restorations showed lesser longevity and higher secondary caries rates compared to amalgam restorations, and this was echoed in the study published in the *Evidence-Based Dentistry* journal [16]. A 2023 study in the U.S. [17] showed that the rate of amalgam restorations declined from a mean of 6.29 per 100 patients in 2017 to 4.78 per 100 patients in 2019, while composite resin restorations increased from 27.6 per 100 patients in 2017 to 28.8 per 100 in 2019. The mean number of amalgam restorations placed per person was lower in females than males. Another study [18] in 2023 indicated that non-amalgam restorations were the most common in the primary teeth of children older than 5 years and in the permanent teeth of adults younger than 40 years.

It seems as if the question of the survival of amalgam versus composite resin has no universally accepted answer; yet, based on the best available evidence, the International Association for Dental Research affirms the safety of dental amalgam while also supporting the phase-down strategy [18].

Our study is based on a large data set, and it focuses on the continuing amalgam longevity debate.

## 2. Materials and Methods

This retrospective study used data from the computerized database of Maccabi Dent, the second-largest dental healthcare provider in Israel, with data on approximately 650,000 dental patients out of the 2.5 million members of the Maccabi Health Fund. This database includes all the information on dental treatments and medical data from Maccabi Dent’s nationwide dental clinics since 2014.

In addition to the data regarding dental treatments, where each procedure has a unique identifying code, data regarding the age and gender of the patient and the clinic location (an indication of socioeconomic status) were also examined. Only restorations on posterior teeth, i.e., molars and premolars, were studied as amalgam is not used as a restorative material in anterior teeth.

### Restorations and Failure File Data

First, all the data on composite resin and amalgam restorations made between 2014–2022 were taken according to the clinical codes of all restorations in both groups. These data are the total base data set.

After that, all the clinical codes belonging to the complications of the restorations in the two experimental groups were collected: all the tooth extractions performed after restoration, the root canals performed after restoration, and the repeated restorations after the failure of a restoration performed between the years 2014–2021 (the file runs up to November 2021). This work file contains the codes of tooth extractions, root canals, and whether amalgam or composite resin fillings were used. It contains 1,954,729 data points, of which the total base data file of the fillings from both groups initially contained 1,547,817 data points.

In the next step, the following exclusion criteria were established (i.e., these types of data were deleted): missing data; data from the year 2022 (in light of the lack of this data in the accompanying file); data on children under the age of 12; and lines with missing numbers of the teeth or numbers of teeth that were not included in one of the 16 relevant numbers (i.e., molar teeth n. 6&7, premolar teeth n. 4&5, and anterior teeth were not included in this study). Double fillings were performed in the same tooth; at the same time, only one filling was taken. Fillings that were conducted on the day the first filling was performed (different codes) and were deleted were not considered a “failure”. Eleven thousand one hundred two fillings that were carried out in teeth where there was a previous record of extraction or a root canal treatment, these were deleted from the data set. After the filtering phase of the initial fillings, the file contained 1,136,034 first fillings, which serves as the relevant database eligible for this study. The failure file contains 131,779 failed fillings and the follow-ups after failure.

To create the file of failures, three types of follow-up treatments that indicated restoration failure were included: the repetition of any restoration, performing a root canal, and tooth extraction. We analyzed the proportions of these follow-up treatments after amalgam and composite restorations.

Treatments were conducted after the first filling was taken from the additional treatments, such as tooth extractions, root canal treatment, composite resin, or amalgam restorations. The period for defining a failure was four years after the restoration. The first restorations were performed in 2014–2018. There were data only until the end of 2021. That mean then, for procedures in 2018, no failures have been recorded yet. An interval of 4 × 365 days was taken.

To determine the relationship between the restoration size and failure rate, we considered the five surfaces of the tooth (occlusal, buccal, lingual, mesial, and distal) and classified the original restorations as follows:

1. Single-surface restoration.
2. Restoration of 2–3 surfaces.
3. Restoration of 4–5 surfaces.
4. Two restorations on the same tooth.

Generalized Estimating Equation (GEE) analysis was performed to assess the associations between the treatment type and failure rate, and adjustments were performed for the potential confounding variables and clustering effects caused by repeated observations within the same subject. A robust estimator covariance matrix, an exchangeable correlation matrix, and a binary logistic model were used. These parameters allow for a fit with the binary outcome, and they assume that the within-subject correlation is constant across all pairs of observations. They also use robust standard errors in the estimation to address potential violations of the data’s assumption of independence or heteroscedasticity. This analysis helps ensure that the statistical inference is valid and reliable, even in correlated or heteroscedastic data.

The results extracted from the analysis were the odds ratio (OR), which represents the likelihood of failure of one treatment type compared to the reference treatment type; and the confidence interval (CI), which provides a range of values within which the true odds ratio is likely to fall.

IRB: This study was approved by the ethics committees of (0002-21 ASMC, 0019-22 MHC).

## 3. Results

Maccabi Dent’s data include information from over 650,000 patients, and 260,905 patients (146,315 females; 114,590 males) were treated between 2014–2021. Of the 260,905 patients in the cohort, 158,940 had repeated treatments.

**Figure 1:**
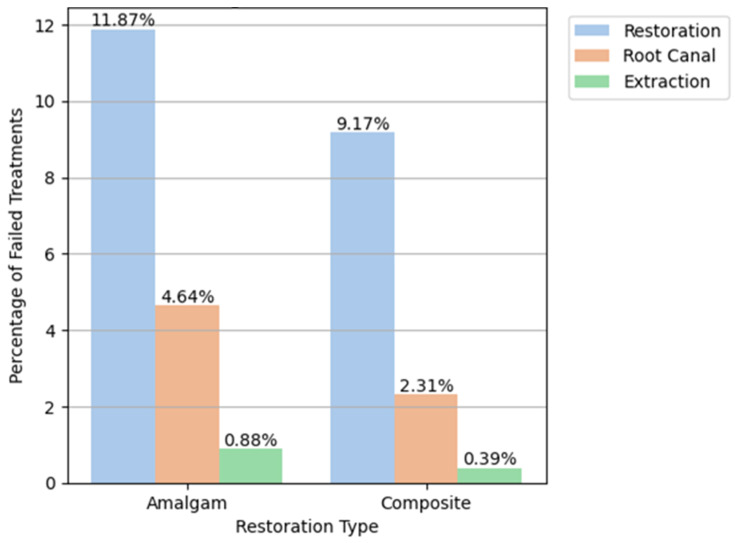
The initial restorative material (amalgam or composite) and subsequent treatment.

The present study had three major outcomes: amalgam, composite, or mixed re-restoration, root canal treatment, or extraction. Figure 1 illustrates the restoration failure and includes the initial restoration treatment code (amalgam or composite), as well as the subsequent treatments on the same tooth, i.e., amalgam, composite, or mixed restoration, root canal treatment, or extraction. It should also be noted that the re-restoration after an initial restoration does not mean that the same restoration type was placed.

Table 1 shows the analysis of the odds ratio (OR), and all comparisons were statistically significant regarding the main effect size (OR = 1.25).

As seen in Table 2, 668,952 restorations were performed for the first time on molars or premolars, of which 113,281 (16.9%) were amalgam, 555,671 (83.1%) composite resin, and 85,635 (12.8%) were categorized as failures. An analysis of the failure percentage based on treatment type revealed a significantly higher percentage of failed amalgams compared to composite restorations in all of the years examined. Specifically, 19,692 out of 113,281 first amalgam restorations failed (17.38%) in all years, whereas 65,943 out of 555,671 composite restorations failed (11.87%) in all years. It is interesting to note that the total number of amalgam restorations decreased in this period while the number of composite fillings increased (Table 1).

Examining the follow-up treatments indicative of amalgam restoration failure revealed that the portion of a repeat restoration was the largest, with 13,442 repeated restorations (11.87% failure), 5252 root canals (4.64%), and 998 extractions (0.88%). The follow-up treatments indicative of composite restoration failures showed that 50,935 (9.17% failure) underwent repeated restoration, 12,821 required root canal treatment (2.31%), and 2187 (0.39%) required extraction (Table 3, Figure 1).

**Figure 2:**
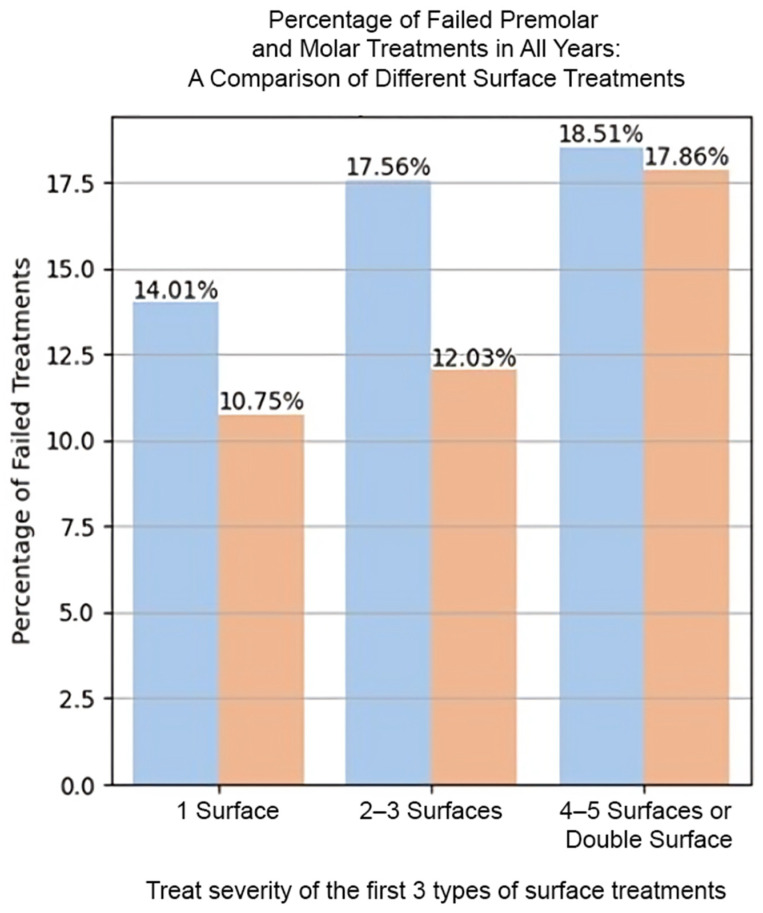
Restoration failure rates based on the number of surfaces treated.

When examining the failure rates of the amalgam and composite restorations based on the number of surfaces involved in the initial restoration, single-surface amalgam restorations failed 14.01% of the time compared to 10.75% in composite. For restorations with 2–3 or and 4–5 surfaces, the failure rate was 17.54% and 18.51% in amalgam compared to 12.07% and 17.86% in composite resin, respectively (Figure 2).

**Figure 3:**
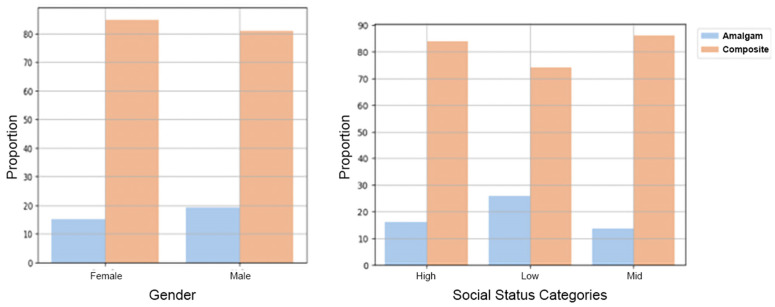
Proportion of the amalgam and composite restorations according to gender and socio-economic status.

There was significantly less amalgam than composite restorations in males compared to females (*p* < 0.0001), as well as in individuals of all socio-economic statuses (*p* < 0.0001) (Figure 3).

Our study concluded that the failure rate over the study period was 17.38% for amalgam restorations and 11.87% for composite resin restorations. The Mean Annual Failure Rate (mAFR) was 3.55% for amalgam restorations and 3.06% for composite resin restorations (*p* < 0.05) (Table 4).

## 4. Discussion

The results of the present study demonstrate a higher survival rate of composite compared to amalgam restorations. These results are based on the data from Maccabi Dent, the second largest HMO in Israel, who have treated more than 650,000 patients in 58 nationwide dental clinics with 440 dental units. In our eight-year retrospective filling-survival cohort, composite restorations showed less failures than amalgam restorations. In the last twenty years, recommendations have been made to reduce the use of mercury. International bodies such as the World Health Organization (WHO) have made similar recommendations in 1997 [19] and in 2021 [20].

Prospective RCT studies have shown equal survival rates for composite and amalgam fillings [21]. In contrast, some retrospective studies have indicated that amalgam is advantageous for large posterior restorations and that posterior composite restorations demonstrate lower survival rates [22]. The different results of the studies can be explained by the research models employed. One of the shortcomings inherent to RCT studies is the lack of long-term follow-up, these studies are often carried out in uniform groups of low-risk participants such as dental teams. These groups are not representative and therefore the findings can have errors due to bias. This shortcoming could be overcome by using a pragmatic research approach. According to Opdam et al. [23], in retrospective practice-based studies, the differences in reconstruction survivals appear after five years, and then a real-life study is required. The studies that meet these requirements are big data studies, such as the current mega-data study that was conducted with data from over eight years.

Some of the previous studies categorized types of restoration failure such as restoration fracture or secondary caries. In the current study, we compared the survival of two restoration materials based on the need for a renewal of a restoration for any reason, root canal treatment, or extraction of the same tooth (the reason for subsequent treatment was not examined).

A recent JADA [24] publication based on 38 trials that evaluated the effectiveness of direct restorative materials to treat caries lesions noted there was limited evidence of clinically important differences between the restorative materials they assessed.

Several studies [25,26,27,28,29] have found that women prefer composite restorations, whereas men prefer amalgam restorations. They have also noted a preference for composite restorations, which are generally more expensive in areas with higher socioeconomic status, while amalgam (which is generally less expensive) is preferred amongst those of a lower socioeconomic status. In the current study, examinations by gender and socioeconomic status revealed a statistically significant preference for composite over amalgam restorations. The large size of our cohort minimizes the chance of random findings. This study shows that restoration failures reduced year on year from 2014 to 2021. We might be able to explain this finding by the improvement in both materials and working methods. Nevertheless, this finding needs further research.

## 5. Limitations

The primary limitation inherent in this study pertains to the potential for selection bias. As a retrospective investigation, this study relies on the analysis of pre-existing data, thus introducing the possibility of inherent biases in the selection of subjects. Furthermore, a notable deficiency arises from the absence of clinical protocols for both procedures, amalgam, and composite resin restorations, which may have led to variations in treatment approaches and outcomes. Additionally, the utilization of dental dams during restorations remains uncertain, introducing further variability into the procedures. Another constraint emerges from the lack of documentation regarding the initial filling’s etiology, thereby potentially influencing survival analyses. Nonetheless, the considerable data set used, comprising 158,940 instances of repeated treatments across both groups, provides a degree of confidence in the relative parity of cause distribution.

Numerous variables have the potential to influence treatment outcomes, including the types of composites employed and the techniques utilized for their placement (e.g., etch and rinse versus self-etch techniques). Moreover, the involvement of multiple dentists across various clinics introduces additional complexity to the analysis. Notably, the failure reasons should have been meticulously recorded in the clinical notes, which would have facilitated a more accurate categorization of treatment failures.

## 6. Conclusions

The current study found no evidence of the superiority of amalgam over composite restorations. At a time when a global environmental decision has been made to reduce the use of mercury, it seems that there are no clinical reasons to continue treatments with amalgam alloy in dentistry. We found that more composite restorations are being placed; this may be due to aesthetic concerns, generally higher compensation, or because some healthcare providers believe that composites are safer. This study, based on big data, indicates that composite restorations have a lower replacement rate than amalgams, and there is now strong evidence to end the long-standing debate on the longevity of amalgam and composite materials. Hence, we can strongly support the Minamata recommendations for the use of composite resin materials only.

## Figures and Tables

**Table 1:** Comparisons between the amalgam and composite failures.

Comparison between Amalgam to Composite Failure Rate According to Subset	Odds Ratio	95% Confidence Interval	*p* Value
Main effect for treatment type	1.255	(1.231,1.279)	<0.0001
Premolar upper jaw	1.650	(1.562,1.743)	<0.0001
Molar upper jaw	1.641	(1.587,1.697)	<0.0001
Premolar lower jaw	1.850	(1.739,1.968)	<0.0001
Molar lower jaw	1.704	(1.651,1.760)	<0/0001
1 surface	1.500	(1.404,1.601)	<0.0001
2–3 surfaces	1.804	(1.759,1.850)	<0.0001
4–5 surfaces/double	1.182	(1.071,1.306)	<0.0001
Upper jaw	1.796	(1.742,1.842)	<0.0001
Lower jaw	1.887	(1.831,1.945)	<0.0001
Premolar	1.789	(1.714,1.867)	<0.0001
Molar	1.698	(1.656,1.740)	<0.0001

**Table 2:** Failure percentage based on treatment type and year.

Treatment Type	First	N Restoration	N Fail	Percentage
Amalgam	2014	21,869	5085	23.25
Composite	2014	46,272	10,412	22.50
Amalgam	2015	19,452	4352	22.37
Composite	2015	47,798	10,101	21.13
Amalgam	2016	18,454	3809	20.64
Composite	2016	59,069	11,571	19.58
Amalgam	2017	15,174	2744	18.08
Composite	2017	67,812	10,942	16.13
Amalgam	2018	13,498	1850	13.70
Composite	2018	73,396	9098	12.39
Amalgam	2019	10,236	1097	10.71
Composite	2019	83,353	7687	9.22
Amalgam	2020	7612	558	7.33
Composite	2020	81,902	4274	5.21
Amalgam	2021	6986	197	2.81
Composite	2021	96,069	1858	1.93
**All**	**all years**	** 668,952 **	** 85,635 **	** 12.80 **
**Amalgam**	**all years**	** 113,281 **	** 19,692 **	** 17.38 **
**Composite**	**all years**	** 555,671 **	** 65,943 **	** 11.86 **

**Table 3:** Follow-up treatments indicative of restoration failure.

1st Treatment Type	2nd Treatment Type	N Fail	Mean	Std
Amalgam	Extraction	998	32.379679	23.606581
Amalgam	Restoration	13,442	31.524709	23.437284
Amalgam	Root Canal	5252	26.046925	22.158989
Composite	Extraction	2187	27.207901	21.948151
Composite	Restoration	50,935	27.187016	21.284104
Composite	Root Canal	12,821	19.715014	19.761084
Amalgam	All Second Treatments	19,692	30.107074	23.24094
Composite	All Second Treatments	65,943	25.734961	21.223939

**Table 4:** The Mean Annual Failure Rates and failure percentage at eight years for restoration type, tooth type, number of surfaces, and follow-up treatment.

Restoration Type	Failure % at Eight Years		Mean Annual Failure Rates (%)	
Factor	Amalgam	Composite	Amalgam	Composite
**Surfaces:**				
1 Surface	14.01	10.75	2.87	2.58
2–3 Surfaces	17.56	12.03	3.39	2.84
4–5 Surfaces/Double Restoration	18.51	17.86	4.01	4.81
**Teeth Group:**				
Molar	17.54	12.07	3.57	3.16
Molar Upper Jaw	16.39	11.36	3.29	3.03
Molar Lower Jaw	18.82	12.68	3.89	3.26
Premolar	16.75	11.60	3.45	2.94
Premolar Upper Jaw	15.51	11.02	3.14	2.82
Premolar Lower Jaw	18.59	12.40	3.91	3.11
**Follow-Up Indicative Treatment:**				
Repeated Restoration	11.87	9.17	2.22	2.27
Root Canal	4.64	2.31	1.05	0.63
Extraction	0.88	0.39	0.18	0.10
**All**	**17.38**	**11.87**	**3.55**	**3.06**

## Data Availability

The data presented in this study are available on request from the corresponding author due to privacy restrictions.

## References

[1] Shenoy A. (2008). Is it the end of the road for dental amalgam? A critical review. J. Conserv. Dent..

[2] Feldbau E.V., Frustino J.L., Migliorini S.A., Sonis S.T. (2015). Chapter 8—Restorative Dentistry. Dental Secrets.

[3] Bharti R., Wadhwani K.K., Tikku A.P., Chandra A. (2010). Dental amalgam: An update. J. Conserv. Dent..

[4] Chadwick R.G., Lloyd C.H. (2022). Dental amalgam: The history and legacy you perhaps never knew?. Br. Dent. J..

[5] Leinfelder K.F. (1991). Dental amalgam alloys. Curr. Opin. Dent..

[6] Marshall S.J., Marshall G.W. (1992). Dental amalgam: The materials. Adv. Dent. Res..

[7] ADA council on Scientific Affairs (1998). Dental amalgam: Update on safety concerns. J. Am. Dent. Assoc..

[8] Bellinger D.C., Daniel D., Trachtenberg F., Tavares M., McKinlay S. (2007). Dental amalgam restorations and children’s neuropsychological function: The New England Children’s Amalgam Trial. Environ. Health Perspect..

[9] DeRouen T.A., Martin M.D., Leroux B.G., Townes B.D., Woods J.S., Leitão J., Castro-Caldas A., Luis H., Bernardo M., Rosenbaum G. (2006). Neurobehavioral effects of dental amalgam in children: A randomized clinical trial. JAMA.

[10] Henshaw D.L., O’Carroll M.J. (2008). European Commission: Scientific Committee on Emerging and Newly Identified Health Risks. The Safety of Dental Amalgam and Alternative Dental Restoration Materials for Patients and Users.

[11] Minamata Convention on Mercury United States Environmental Protection Agency. https://www.mercuryconvention.org/sites/default/files/2021-06/minamata-convention-booklet-sep2019-en-pdf.

[12] Estrich C.G., Lipman R.D., Araujo M.W.B. (2021). Dental amalgam restorations in nationally representative sample of US population aged ≥15 years: NHANES 2011–2016. J. Public Health Dent..

[13] Khangura S.D., Seal K., Esfandiari S., Quiñonez C., Mierzwinski-Urban M., Mulla S.M., Laplante S., Tsoi B., Godfrey C., Weeks L. (2018). Composite Resin Versus Amalgam for Dental Restorations: A Health Technology Assessment [Internet].

[14] Rasines Alcaraz M.G., Veitz-Keenan A., Sahrmann P., Schmidlin P.R., Davis D., Iheozor-Ejiofor Z. (2014). Direct composite resin fillings versus amalgam fillings for permanent or adult posterior teeth. Cochrane Database Syst. Rev..

[15] Worthington H.V., Khangura S., Seal K., Mierzwinski-Urban M., Veitz-Keenan A., Sahrmann P., Schmidlin P.R., Davis D., Iheozor-Ejiofor Z., Rasines Alcaraz M.G. (2021). Direct composite resin fillings versus amalgam fillings for permanent posterior teeth. Cochrane Database Syst. Rev..

[16] Maciel C.M., Baroudi K., Costa L.D.C., Souto T.C.V., Pino Vitti R. (2022). Longevity of Resin Composite and Amalgam Posterior Restorations: A Systematic Review. Eur. J. Prosthodont. Restor. Dent..

[17] Bernardo M., Luis H., Martin M.D., Leroux B.G., Rue T., Leitão J., DeRouen T.A. (2007). Survival and reasons for failure of amalgam versus composite posterior restorations placed in a randomized clinical trial. J. Am. Dent. Assoc..

[18] Alhareky M., Tavares M. (2016). Amalgam vs Composite Restoration, Survival, and Secondary Caries. J. Evid. Based Dent. Pract..

[19] Estrich C.G., Eldridge L.A., Lipman R.D., Araujo M.W.B. (2023). Posterior dental restoration material choices in privately insured people in the United States, 2017 through 2019. J. Am. Dent. Assoc..

[20] Beltrán-Aguilar E.D., Thornton-Evans G., Wei L., Bernal J. (2023). Prevalence and mean number of teeth with amalgam and nonamalgam restorations, United States, 2015 through 2018. J. Am. Dent. Assoc..

[21] Ajiboye A.S., Mossey P.A., Fox C.H., IADR Science Information Committee (2020). International Association for Dental Research Policy and Position Statements on the Safety of Dental Amalgam. J. Dent. Res..

[22] FDI World Dental Federation (2024). Alternative direct restorative materials to dental amalgam. Int. Dent. J..

[23] Minamata Convention on Mercury (2021). Report 197, Commonwealth of Australia. https://www.dcceew.gov.au/environment/protection/chemicals-management/mercury.

[24] Manhart J., Chen H., Hamm G., Hickel R. (2004). Buonocore Memorial Lecture. Review of the clinical survival of direct and indirect restorations in posterior teeth of the permanent dentition. Oper Dent..

[25] Van Nieuwenhuysen J.P., D’Hoore W., Carvalho J., Qvist V. (2003). Long-term evaluation of extensive restorations in permanent teeth. J. Dent..

[26] Opdam N.J., Bronkhorst E.M., Cenci M.S., Huysmans M.C., Wilson N.H. (2011). Age of failed restorations: A deceptive longevity parameter. J. Dent..

[27] Pilcher L., Pahlke S., Urquhart O., O’Brien K.K., Dhar V., Fontana M., González-Cabezas C., Keels M.A., Mascarenhas A.K., Nascimento M.M. (2023). Direct materials for restoring caries lesions: Systematic review and meta-analysis-a report of the American Dental Association Council on Scientific Affairs. J. Am. Dent. Assoc..

[28] Willershausen B., Witzel S., Schuster S., Kasaj A. (2010). Influence of gender and social factors on oral health, treatment degree and choice of dental restorative materials in patients from a dental school. Int. J. Dent. Hyg..

[29] Bailey O., Stone S., Ternent L., Vernazza C.R. (2022). Public Valuation of Direct Restorations: A Discrete Choice Experiment. J. Dent. Res..

